# Selenium as a Modulator of Reproductive Immunity: Molecular Insights and Translational Potential in Livestock

**DOI:** 10.1111/rda.70146

**Published:** 2025-11-21

**Authors:** Muhammad Usman, Riffat Maqsood, Roshan Riaz, Idil Şerbetçi, Muhammad Nasir Bhaya, Mahmood Ul Hassan

**Affiliations:** ^1^ Faculty of Veterinary Science University of Agriculture Faisalabad Faisalabad Pakistan; ^2^ Department of Animal Nutrition and Nutritional Diseases, Faculty of Veterinary Medicine Kafkas University Türkiye; ^3^ Republic of Türkiye Ministry of Agriculture and Forestry General Directorate of Agricultural Research and Policies, International Center for Livestock Research and Training Ankara Türkiye; ^4^ Department of Pathology, Faculty of Veterinary Science University of Agriculture Faisalabad Faisalabad Pakistan; ^5^ Department of Agricultural, Food and Forestry Science (SAAF) University of Palermo Palermo Italy

**Keywords:** antioxidant, immunity, livestock, reproduction, selenium

## Abstract

The periparturient period represents a critical window of vulnerability in livestock reproduction. Additionally, reproductive performance is often compromised due to a weakened immune system and high oxidative stress. Selenium, an essential micronutrient, emerges as a key element with dual roles in antioxidant defence and immune modulation, making it a cornerstone in maintaining reproductive health in livestock. Selenium exerts its protective effects through incorporation into selenoproteins such as glutathione peroxidase (GPx), which downregulate oxidative stress, support cellular integrity, and regulate inflammation in reproductive tissues. During the periparturient period, selenium deficiency is associated with increased production of β‐hydroxybutyric acid (BHBA) and non‐esterified fatty acids (NEFA), responsible for triggering lipid mobilisation and activation of the NF‐κB (Nuclear Factor kappa‐light‐chain‐enhancer of activated B cells) signalling pathway. This leads to overexpression of pro‐inflammatory genes, resulting in uterine infections, mastitis, and other reproductive disorders. Selenium supplementation in organic or nano forms plays a potential role in countering these effects by activating the Nrf2 (Nuclear factor erythroid 2) pathway, boosting antioxidant enzymes, and suppressing the NF‐κB pathway. In females, selenium enhances endometrial epithelial repair, hormone regulation, and immune tolerance by regulating the NF‐κB signalling pathway. In males, combined supplementation of selenium with vitamin E improves sperm quality, motility, and testosterone levels while preventing lipid peroxidation in spermatozoa. At the epigenetic level, selenium influences histone acetylation to regulate transcription of inflammatory genes such as COX‐2 and TNF‐α. Recent insights into the role of selenium receptors (LRP8) in ovarian follicular development highlight the applications of selenium in fertility regulation. The efficacy of selenium is highly influenced by its form, dosage, animal species, and physiological state. This review emphasises the need for large‐scale, species‐specific research trials, nanodelivery strategies, and omics‐based biomarkers to improve selenium supplementation strategies and dose rate. Selenium holds significant translational potential in veterinary reproduction, playing a preventative and therapeutic role against reproductive immunopathologies in livestock.

## 1 Introduction

Reproduction and fertility of animals are greatly associated with reproductive immunity, especially during the periparturient period. Reproductive immunity is the interaction between the immune and reproductive systems, crucial for the fertility of animals. The delicate balance between immune responses and immune tolerance should be maintained during the periparturient period for successful reproduction in the future. During this period, any disruption in this balance increases susceptibility to infections due to a compromised immune response. These infections may prove to be highly crucial for reproduction and fertility by causing embryonic loss, metritis, and mastitis (Khan et al. 2024). The immune system is directly supported by balanced nutrition, especially during transition periods. A balanced nutrition containing essential micronutrients like selenium (Se), copper (Cu), and zinc (Zn) ensures the normal birth of healthy offspring and increases fertility by reducing the incidence of disease (Van Emon et al. 2020). Recent research has revolutionised the importance of micronutrients like selenium, vitamin E, copper, zinc and manganese. The combined supplementation of selenium and vitamin E plays a pivotal role in reducing the incidence rate of retained placenta and improving immune function. But the combined supplementation of copper, zinc, and manganese regulates milk yield and energy production (Zheng et al. 2022). Selenium is one of the obligatory micronutrients playing crucial roles in various reproductive physiological processes by preventing oxidative stress to cells (Al‐Saeed et al. 2023). In addition, it plays a crucial role in immune modulation by acting as a cofactor for selenoproteins (Hosseintabar‐Ghasemabad et al. 2024; Sadler et al. 2024). Selenium plays a potential role in spermatogenesis, synthesis of reproductive hormones, and steroidogenesis in animals by reducing oxidative stress on cells (Vaswani and Kumar 2023). Furthermore, it enhances the fertility of livestock by reducing the incidence of reproductive problems by enhancing immune responses (Pecoraro et al. 2022). Selenium has an influential role in innate and adaptive immunity by modulating the activity of selenoenzymes, which control cytokine production and inflammatory responses. Recent research has demonstrated that selenium supplementation plays a critical role in improving the efficiency of the reproductive system by increasing conception rates and improving estrus cyclicity in ruminants. Benitez has demonstrated the effect of oral selenium supplementation on the reproductive tract in rams. Inorganic selenium improves reproductive efficiency by increasing sperm motility, volume, concentration and quality by increasing testosterone levels in the body (Muñoz‐Benitez et al. 2025).

The role of selenium in veterinary reproduction and immunology crucially supports its use for enhancing the fertility of animals. The individual roles of selenium in animal reproduction and immunology are well recognized. But there is still a need for comprehensive advancements and reviews on exploring the role of selenium in modulating the reproductive immunity during crucial reproductive stages. There is a great need for immunity in the female reproductive tract due to high susceptibility to infections during the periparturient period. The animal trials and research are consistently challenged by the environmental factors and heat stress leading to reproductive problems (Zheng et al. 2022). The reproductive outcomes in livestock are optimized by good nutritional strategies. This review explores and highlights the molecular mechanisms of selenium in reproductive immunity to increase fertility rates in livestock.

## 2 Selenium: Biochemistry and Immunological Role

Selenium is broadly classified into organic and inorganic categories for their supplementation to livestock. Oral supplementation of inorganic selenium undergoes necessary changes to convert into organic selenium for potential benefits, efficient absorption, more bioavailability, and reduced toxicity (Yuan et al. 2024). The organic form of selenium usually includes selenomethionine and selenocysteine, which are substituents of methionine and cysteine in proteins, respectively (Huang et al. 2025). These forms of selenium are extracted from animals and plants. But the inorganic forms of selenium are usually selenate (SeO_4_^2−^) and selenite (SeO_3_^2−^), which are commonly present in soil and water (Azorín et al. 2024). Selenium plays a crucial role as a precursor for the production of selenoproteins, depending on its bioavailability. Selenoproteins are produced from certain selenoprotein genes having incorporated selenium in the form of selenocysteine (Khan et al. 2024). Selenoproteins like glutathione peroxidases (GPx‐1, GPx‐3 and GPx‐4) (Akpinar et al. 2023), selenoprotein P (SELENOP), and selenoprotein S (SELENOS) play a crucial role in sperm development, maturation, protection of ovarian follicles, and regulating oophoritis and placentitis. Glutathione peroxidase (especially GPx4) is one of the selenoproteins well characterized for reducing oxidative stress to cells by utilizing glutathione as a substrate and catalyzing the reduction of hydrogen peroxide and lipid hydroperoxides. As an immune modulator, selenium plays an influential role in both innate and adaptive immunity. Selenium supplementation is greatly associated with the regulation of T‐cell proliferation, NK cell activity, and production of cytokines (Méndez López et al. 2024). So the deficiency of selenium may lead to oxidative stress, impaired immune responses, and ferroptosis, resulting in various diseases (Shen et al. 2022; Sun et al. 2023).

## 3 Reproductive Immunity: An Overview

The reproductive system of livestock is compromised by some unique immunological challenges during pregnancy and after calving. During pregnancy, the maternal immune system is highly regulated for the safe development of the fetus in utero. The maternal immune system shifts from Th1 to Th2 responses during pregnancy (Trevisi et al. 2025). These high levels of Th2 responses in the maternal body favor humoral immunity over cell‐mediated immunity. During this period, the dam is highly susceptible to infections due to reduced cell‐mediated immunity. While after parturition, the maternal immune system starts shifting toward Th1 responses and changes to cell‐mediated immunity. But the transition from Th2 to Th1 is not immediate and needs time for its recovery. During its recovery, the immune system of a dam experiences a temporary period of immune suppression called periparturient immunosuppression (Brodzki et al. 2024). During this period, the dam is highly susceptible to uterine infections like metritis and may lead to infertility by producing antisperm antibodies (Gupta et al. 2023).

The presence of pathogens in the reproductive tract may induce immune responses leading to severe inflammation. Severe inflammation without recovery in the reproductive tract may result in the permanent loss of fertility. In this regard, some organs of the reproductive tract, like the testes, uterus and placenta, are considered as immune privilege sites for the protection of gametes and fetus from intense immune response (Yüzen et al. 2022). These immune privilege sites prevent intense immune responses by tolerating antigens through various mechanisms, such as increased immunoinhibitory substances like 19‐hydroxy prostaglandin E and polyamines in seminal plasma and Toll‐like receptors on endometrial epithelium (Gupta et al. 2023). The fetus is prevented from maternal immune responses by the accumulation of regulatory T‐cells and mucosal immunity in the uterus. Excessive regulatory T cells in the uterus play a critical role in promoting immune tolerance to fetal antigens (Traxinger et al. 2022). However, the uterine mucosa is greatly associated with the recognition of lipopolysaccharides due to the presence of neutrophils and macrophages (Ault‐Seay et al. 2023) (Table 1).

**TABLE 1:** Tissue‐specific targets of selenium in reproductive immunity.

Target tissue	Primary immune role of selenium	Selenium‐linked activity	Associated pathways	References
Uterine epithelium of cows	Defence during postpartum period	Anti‐inflammatory role against LPS; boosts epithelial repair	Low NF‐κB, high GPx1 and Nrf2	(Cui et al. 2023)
Leydig & Sertoli cells	Hormone synthesis, immune privilege, spermatogenesis	Protects testicular environment from ROS; supports testosterone synthesis	Low caspase‐3 and high GPx4, steroidogenic acute regulatory protein (StAR)	(Cui et al. 2023)
Placental trophoblasts in goats	Immune barrier and nutrient transfer to fetus	Selenium modulates cytokine balance, supports fetal‐maternal tolerance	Low IL‐1β, and high IL‐10, TrxR, and GPx4	(Yuan et al. 2025)
Spermatozoa of bulls and rams	Fertilisation capacity	Protects against DNA fragmentation and lipid peroxidation	High SOD, and GPx, low MDA	(Li et al. 2023)
Colostrum of cows and goats	Transfer of passive immunity to neonates	Increases IgG, Se concentration in colostrum	High IgG, and Se‐binding proteins	(Erickson 2022)

## 4 Selenium and Female Reproductive Immunity

The incidence rates of infections in the female reproductive tract of livestock are relatively high during the periparturient period due to immunosuppression. The infections may be due to high levels of lipopolysaccharide and cortisol in the female reproductive tract of domestic animals. Se supplementation plays a crucial role in mediating the inflammatory responses due to high levels of LPS and cortisol (Li, Dong et al. 2024). Se enhances the proliferation of endometrial epithelium and suppresses apoptosis by stimulating the expression of key genes involved in the induction of PI3K/AKT/GSK‐3β and Wnt/β‐catenin signalling pathways (Dong et al. 2024). The expression of growth factor genes like TGFB1, TGFB3, and VEGFA is also induced with selenium supplementation, resulting in the improved blood vessel repair and controlled inflammation (Li, Wang, et al. 2024). Additionally, the expression of pro‐inflammatory cytokines is also suppressed by the inhibition of NF‐κB and MAPK signalling pathways (Yuan et al. 2025). In this way, Se supplementation prevents interruption in reproduction of domestic animals by regulating the ovarian functioning and hormone levels (Dong et al. 2024).

Recent research has described that selenium supplementation to dairy cows and buffalos plays a potential role in preventing mastitis by fighting *Staphylococcus aureus* during the postpartum period (Khan et al. 2024). Insufficient feed intake during the postpartum period forces the body to break down body fat, resulting in the elevated production of harmful substances like NEFA (non‐esterified fatty acids) and BHBA (β‐hydroxybutyric acid), which indicate mastitis susceptibility (Khan et al. 2022). These substances are responsible for high reactive oxygen species (ROS) leading to mastitis. Selenium supplementation plays a beneficial role in reducing the severity of mastitis by acting as an antioxidant and immune booster. It increases the levels of glutathione peroxidase, which acts as a powerful antioxidant enzyme. Furthermore, it reduces the ROS and inflammatory cytokines in the udder by blocking MAPK and NF‐κB pathways. The use of selenium nanoparticles is highly desirable for better absorption, strong antimicrobial action, and reduced bacterial resistance in the udder.

**FIGURE 1:**
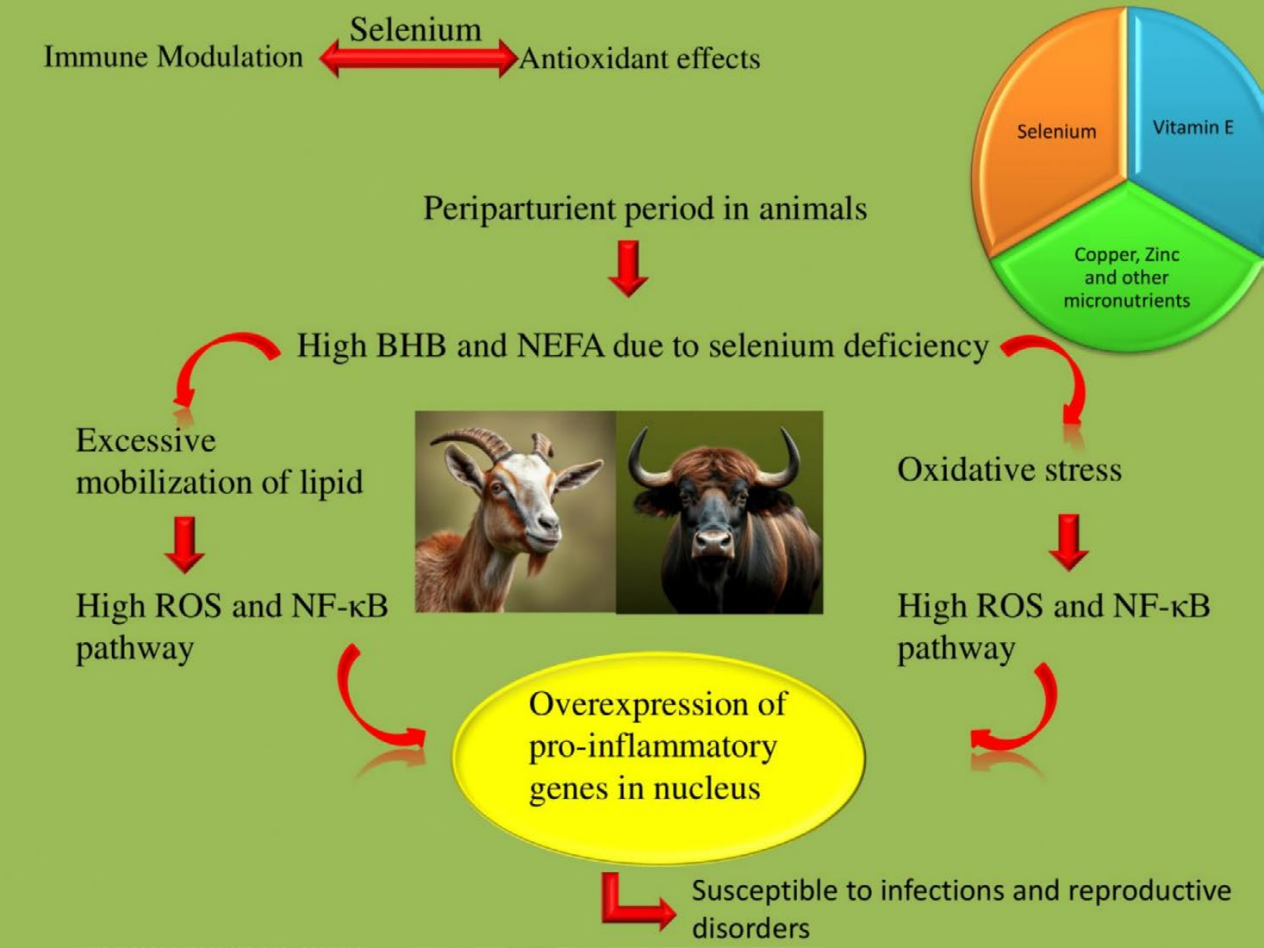
Selenium deficiency during the periparturient period in animals causes metabolic and oxidative stress. This activates the NF‐κB signalling pathway, resulting in inflammation through the overexpression of pro‐inflammatory genes. Excessive inflammation in the female reproductive tract leads to uterine infections. The optimum dose of selenium, along with other antioxidants like vitamin E, is crucial for maintaining health during this period.

Recent studies have described the role of combined supplementation of cortisol with selenium to prevent oxidative stress in bovine endometrial cells due to the exposure of LPS. The presence of LPS in endometrial cells causes oxidative stress, leading to high ROS levels. Selenium and cortisol have a synergistic effect on activating the Nrf2 signalling pathway. The entry of selenium and cortisol into the cell initiates the translocation of Nrf2 into the nucleus, where it binds to antioxidant response elements, resulting in the production of antioxidant enzymes like HO‐1 and NQO‐1. Cortisol administration is highly associated with increased antioxidant levels like Total Antioxidant Capacity (T‐AOC), catalase (CAT), superoxide dismutase (SOD), glutathione peroxidase (GSH‐Px) and Thioredoxin Reductase (TRXR), which are responsible for reduced ROS (Al‐Gheffari et al. 2024). On the other hand, selenium plays a potential role in the upregulation of antioxidants like GSH‐Px and TRXR (Adeniran et al. 2022; Balgoon and Alghamdi 2024). The optimum dose rate for selenium and cortisol is 2–4 μM and 15–30 ng/mL, respectively, for efficient protection against oxidative stress (Cui et al. 2025) (Figure 1).

## 5 Selenium and Male Reproductive Immunity

**FIGURE 2:**
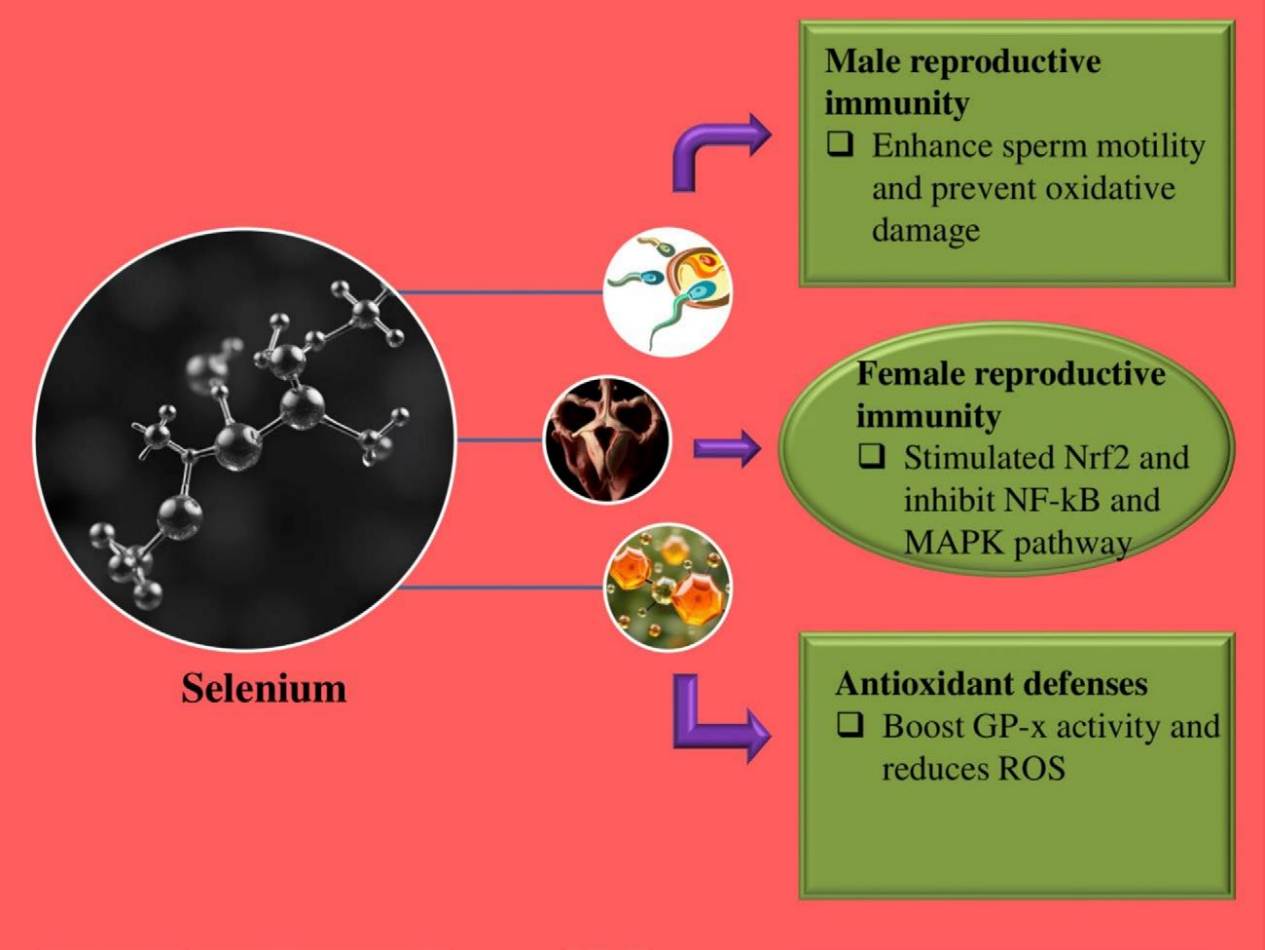
Role of Selenium in reproductive immunity is illustrated (Vaswani and Kumar 2023).

The quality of sperm is highly affected by oxidative stress, leading to the production of abnormal sperm. The sperm membrane is attacked and destroyed by reactive oxygen species during oxidative stress. High levels of reactive oxygen species impair sperm functioning and fertility by resulting in lipid peroxidation, DNA fragmentation, and apoptosis (Mannucci et al. 2022). Selenium supplementation plays a potential role in male fertility by improving the quality of sperm and reducing oxidative stress to sperm. Recent research has revealed the potential role of combined supplementation of selenium and vitamin E in protecting sperm from oxidative damage (Fadl et al. 2024). Selenium detoxifies the reactive oxygen species by boosting the activity of the GPx enzyme and supports the mitochondria and tail of sperm. On the other hand, vitamin E prevents sperm death by regulating the activity of glutathione‐dependent enzymes. The addition of 200 IU of vitamin E and 0.2 mg/kg of selenium in the feed of male animals every week leads to higher sperm motility, normal morphology of sperm, higher testosterone levels and improved testis size. This results in high conception rates at large scale by improving the quality of fresh and frozen semen (Fadl et al. 2024) (Figure 2).

## 6 Molecular and Cellular Mechanisms

Selenium plays a crucial role in regulating the intensity of inflammation in the uterus during the postpartum period by controlling the histone acetylation and regulating signaling pathways in uterine cells. Acetylation of histone proteins causes the loosening of DNA, which triggers the pro‐inflammatory genes, resulting in excessive inflammation in the uterus. Se supplementation in the form of selenite plays a potential role in reducing inflammation in the presence of selenoproteins. It suppresses the acetylation of histone H4 at K12 and K16 by blocking the acetylation‐inducing enzymes like p300 which leads to the tightening of DNA (Narayan et al. 2015). The expression of pro‐inflammatory genes like COX‐2 and TNF‐α is also suppressed by reduced histone acetylation. Additionally, selenium helps the body in the production of many anti‐inflammatory compounds like cyclopentenone prostaglandins (CyPG). These anti‐inflammatory compounds reduce the inflammation by transforming the already present macrophages from the M1 state (fighters) to the M2 state (healers) (Campo‐Sabariz et al. 2022; Sinha et al. 2023). This results in promoting tissue healing and the health of the uterus during critical reproductive stages.

Selenium also regulates the cell signalling pathways, especially the NF‐κB pathway, to reduce inflammation and supports the epithelial barrier in the mammary gland and uterus during crucial reproductive stages (Liu et al. 2025). Selenium inhibits the NF‐κB pathway in order to prevent excessive inflammation in the uterus by inhibiting the phosphorylation of IκBα, resulting in prevention of NF‐κB (p65) entry in the nucleus. The accumulation of NF‐κB (p65) in the cytoplasm reduces the acetylation of p65 at the lysine 310 site, leading to reduced mRNA levels of pro‐inflammatory genes like IL‐1β, IL‐6, iNOS, COX‐2 and TNF‐α (Cui et al. 2023). The administration of selenium nanoparticles has been proved very effective in reducing oxidative damage to tissues by boosting antioxidant defence systems (Yang et al. 2024).

Recent research has also revolutionised the functional antioxidant enzymes and selenium receptors in granulosa cells of ovarian follicles. Granulosa cells of the ovarian follicle are responsible for the nourishment of the egg and production of steroid hormones like oestrogen and progesterone. The presence of steroid hormones may lead to high production of reactive oxygen species, which is harmful for the development of eggs (Hummitzsch et al. 2024). Antioxidant enzymes like GPX1, SOD, CAT, PRDX and TXN play a potential role in preventing the granulosa cells from oxidative stress and promoting egg development. Among many antioxidants, GPX1 has been proven efficient in reducing the oxidative stress to eggs. The presence of selenium receptors like LRP2 and LRP8 on granulosa cells plays a crucial role in selenium uptake from the bloodstream. LRP8 is a selenium uptake receptor responsible for high selenium uptake from blood and results in increased follicular size. LRP8 and GPX1 are the main defenders of granulosa cells, preventing them from oxidative stress (Hummitzsch et al. 2024).

## 7 Nutritional Strategies, Supplementation and Toxicity

The stimulation of reproductive immunity depends upon the forms of selenium supplementation in animals. The supplementation of organic selenium (selenomethionine or Se‐enriched yeast) has shown higher bioavailability and absorption than inorganic selenium (sodium selenite), especially in monogastric animals compared to ruminants (Malyugina et al. 2021). Organic selenium has been proved to be effective in preventing the animal from reproductive disorders by various methods depending upon the type of species of animals. In cattle, organic selenium reduces oxidative stress by boosting antioxidant defense mechanisms and boosts the activity of GSH‐Px enzymes (Huang et al. 2023). Organic selenium administered to sows at the optimal dose rate of 0.5 ppm leads to high levels of selenium in colostrum, and serum (Jin et al. 2022). It also plays a crucial role in improving body weight in piglets during weaning. Furthermore, organic selenium results in improved immune markers for better recognition of pathogens in the reproductive system (Pecoraro et al. 2022).

### 7.1 Selenium Supplementation

Selenium supplementation strategies greatly depend upon the health status of animals. Under normal conditions, organic selenium sources like selenized yeast and Se‐methionine are usually added to the feed of animals. The ideal inclusion levels of selenium are 0.2–0.5 mg Se/kg dry matter of feed to improve the activity of glutathione peroxidase enzyme, resulting in a stimulated antioxidant defence system in poultry and ruminants (Liu et al. 2023). But during susceptible reproductive periods, selenium is supplemented in the form of selenium enriched hay and milk to prevent animals from bacterial infections. It prevents animals from high oxidative stress during the periparturient period by boosting the antioxidant defence system, and immunity (Zhou et al. 2025). Selenium could also be administered to animals in the form of nanoparticles and coated forms during heat stress and other harsh environmental conditions to improve its bioavailability and absorption. It plays a crucial role in enhancing the proportion of heat shock proteins and restoring the microbial balance in the gut by improving rumen fermentation (Li et al. 2023a; Sitaresmi et al. 2024) (Table 2).

**TABLE 2:** Selenium supplementation strategies in livestock, like dosage, outcomes and species are described.

Selenium form	Species	Route	Reproductive outcome	References
Sodium Se + Vit E	Dairy cows	IM or oral	Reduced Mastitis and high plasma GPx	(Vasiľ et al. 2022)
Se + Vit E	Dairy cows	Dietary	Improved neutrophil killing and Reduced Mastitis	(Vasiľ et al. 2022)
Se‐yeast	Dairy cows	Dietary	High IgG in colostrum and blood; faster Se transfer to calf	(Ceballos‐Marquez et al. 2010)
Nano‐Se	Sheep	Dietary	Enhances rumen function and antioxidant enzyme activity	(Rabee et al. 2023)

### 7.2 Selenium Toxicity

Selenium dosage is crucial in the feed of livestock, as there is a narrow margin between beneficial and toxic intake of selenium. Overdosage of selenium may cause chronic or acute poisoning in livestock. Acute selenium poisoning is characterised by anorexia, respiratory distress and death, while chronic selenium poisoning (selenosis) may result in lameness, classical selenosis lesions, reduced milk yield, hoof deformities, and poor reproductive efficiency in males and females. The organic forms of selenium have generally high bioavailability, which allows them to remain inside the body and contribute to chronic selenium toxicity. It may lead to cellular damage by disrupting mitochondrial functioning, reducing sperm quality, embryotoxicity, and impaired spermatogenesis and folliculogenesis, especially in chronic selenium poisoning. But selenium toxicity is usually modulated by the administration of other minerals like sulfur by decreasing selenium absorption and modifying selenium kinetics in the body. Selenium status of the herd should be evaluated regularly by various biomarkers like glutathione peroxidase activity to ensure supplementation remains within a safe and effective range.

## 8 Translational Potential and Future Directions

Selenium supplementation plays a crucial role in the expression of selenoproteins for preventing oxidative stress and supporting reproductive immunity. Selenium deficiencies in animal feed may lead to the suppression of selenoproteins like GPX and TXN. In this way, the selenium status of animals is measured by the levels of selenoprotein gene expression and has been proven to be an effective biomarker to diagnose early reproductive pathologies. Additionally, selenium deficiencies lead to oxidative stress marked by increased oxidative biomarkers and inflammatory cytokines, and iNOS causes potential DNA damage and reduced antioxidant enzymes. These changes critically influence sperm motility, oocyte quality, embryo development and implantation due to lipid peroxidation and ROS. Severe inflammation and apoptosis in uterine cells may lead to the formation of inflammatory lesions and placental inflammation. Thus, this provides the molecular basis for the requirement of selenium supplementation for preventing the reproductive system and gut from various infections (Lei et al. 2023) (Table 3).

**TABLE 3:** Forms of selenium supplementation in livestock reproductive immunity with molecular studies are described.

Selenium form	Species	Outcome	Molecular insight	References
Organic Se (Se‐yeast)	During postpartum in Dairy cows	Reduced LPS‐induced endometritis, improved uterine repair	Inhibited NF‐κB/MAPK, activated Nrf2/PI3K‐AKT/Wnt‐β‐catenin in endometrial cells	(Li, Wang, et al. 2024)
Sodium selenite (inorganic Se)	Bovine IVF oocyte culture	High Embryo competence: enhanced blastocyst rates	Boosted GPx activity in oocytes, improved ROS clearance during IVM	(Qazi et al. 2018)
Sodium selenite (2–4 μM)	Bovine endometrial stromal cells	Reduce LPS and high‐cortisol inflammation, high GPx1/4 expression and cell repair	Suppresses TLR4 → MyD88 → NF‐κB/MAPK, boosts GPx1/4; activated by cortisol	(Cui et al. 2024)
Selenium‐enriched antioxidants (SeNP + GSH)	Bull semen cryopreservation	Less ROS, high post‐thaw viability & blastocyst development	Synergistic protection via GPx and TrxR activation during cryopreservation	(Li et al. 2023)
Se + Vitamin E supplementation	Dairy cows, periparturient period	Less Mastitis incidence, less somatic cell count (SCC); less milk GPx activity	Intensifies antioxidant defence; reduces inflammasome/TLR2‐NF‐κB signalling	(Khan et al. 2024)

Despite progress in understanding the role of selenium in modulating reproductive immunity, there are several critical research gaps hindering the full translational application of selenium in veterinary reproduction. Most of the recent data have been derived from in vitro models or limited animal trials, leaving a pressing need for large‐scale, controlled and species‐specific in vivo research. Additionally, optimal dosing strategies, specific molecular targets like serum concentration of catalase, and comparative efficacy of various selenium forms (organic, inorganic, nano‐selenium) across different reproductive states remain underexplored (Saadullah et al. 2024; Vaswani and Kumar 2023). Many emerging technologies, like nanodelivery systems and multi‐omics biomarker profiling for early diagnosis of reproductive pathologies, play an important role in advancing selenium‐based interventions (Dong et al. 2024). But their practical use in herd health management requires rigorous testing and validation, leading to large expenses. Addressing these gaps is essential to understanding the potential of selenium as a diagnostic and therapeutic tool in reproductive health in livestock.

## 9 Conclusion

Selenium plays a crucial role in regulating reproductive immunity in livestock by reducing oxidative stress and modulating immune responses during the periparturient period. Due to its involvement in antioxidant defense and regulation of key signaling pathways like NF‐κB and Nrf2, it is responsible for supporting both male and female fertility. Selenium supplementation regulates sperm quality and uterine health and supports hormonal balance during crucial reproductive stages. Organic and nano‐selenium forms of selenium offer better bioavailability and efficiency for animals. The combined supplementation of selenium with other antioxidants like vitamin E significantly reduces reproductive disorders such as mastitis, metritis and infertility. Despite the proven benefits, further species‐specific and large‐scale studies are needed to optimize the use of selenium. Selenium holds strong translational potential for improving livestock reproductive outcomes.

## Author Contributions

M.U., R.M. and M.N.B. were responsible for conducting the literature search, analysing the collected records and drafting, with oversight from R.R., M.U.H. and I.Ş. critically evaluated the literature, contributed to the synthesis of evidence, assessed the quality of sources and assisted in drafting and refining the manuscript. All authors reviewed and approved the final version of the manuscript.

## Conflicts of Interest

The authors declare no conflicts of interest.

## Data Availability

Data sharing not applicable to this article as no datasets were generated or analyzed during the current study.
